# Testosterone is associated with abdominal body composition derived from computed tomography: a large cross sectional study

**DOI:** 10.1038/s41598-022-27182-y

**Published:** 2022-12-29

**Authors:** Seungbong Han, Young-Jee Jeon, Tae Young Lee, Gyung-Min Park, Sungchan Park, Seong Cheol Kim

**Affiliations:** 1 Department of Biostatistics, Korea University College of Medicine, Seoul, Korea; 2 Department of Family Medicine, Ulsan University Hospital, University of Ulsan College of Medicine, Ulsan, Korea; 3 Department of Radiology, Ulsan University Hospital, University of Ulsan College of Medicine, Ulsan, Korea; 4 Department of Internal Medicine, Ulsan University Hospital, University of Ulsan College of Medicine, Ulsan, Korea; 5 Department of Urology, Ulsan University Hospital, University of Ulsan College of Medicine, Ulsan, Korea

**Keywords:** Urology, Endocrine system and metabolic diseases

## Abstract

The aim of this study was to evaluate the association between serum testosterone and abdominal body composition based on abdominopelvic computed tomography (APCT) measurements after adjusting for individual metabolic syndrome components. We performed a cross-sectional study using male subjects (age range: 22–84 years) who underwent a general health examination with abdominopelvic computed tomography and testosterone measurements. Body composition was evaluated with APCT. To confirm an association between testosterone and abdominal body composition, we conducted linear regression analysis. The effect of abdominal body composition was adjusted for important clinical factors such as age, albumin, and metabolic components in the multivariable regression analysis. Overall, 1453 subjects were included in the primary analysis. After adjustment for age, individual metabolic components, albumin, hemoglobin A1c, and C-reactive protein, we found that subcutaneous fat area index (β = − 0.042, *p* < 0.001), total abdominal muscle area index (β = 0.115, *p* < 0.001), normal attenuation muscle area index (β = 0.070, *p* < 0.001), and log_e_-transformed lower attenuation muscle area index (β = 0.140, *p* = 0.002) had an association with log_e_-transformed testosterone level. After adjusting for individual metabolic syndrome components, testosterone was associated negatively with subcutaneous fat, but not visceral fat. In addition, testosterone was positively correlated with abdominal muscle regardless of qualitative features such as fat-rich and fat-free.

Korea is rapidly becoming an aging society and aging itself is becoming a global health issue. Body composition can change with aging, which includes decreased skeletal muscle mass and increased abdominal fat mass^1^. The causal relationships between testosterone and the age-related changes have not yet been elucidated; however, some studies have established this relationship^2^. Epidemiological studies have found that lower testosterone levels are related to decreased muscle mass, central obesity, and the accumulation of abdominal fat^3,4^.

Metabolic syndrome (MetS) is characterized by several specific components, including increased waist circumference, dyslipidemia, hypertension, and impaired glucose tolerance. We previously showed that MetS and diabetes were associated with a change in abdominal body composition^5,6^. MetS also has a close relation with low testosterone^7^. Due to the strong correlation that exists among testosterone, MetS, and body composition, MetS should be considered and adjusted in order to evaluate whether testosterone directly affects body composition. However, few studies have evaluated the relationship between testosterone and abdominal body composition after adjusting for MetS. For this reason, the results of previous studies on testosterone and abdominal body composition differed^8^. Therefore, it is necessary to confirm how testosterone directly affects abdominal body composition without secondary effects by MetS.

Computed tomography (CT) can directly measure areas of fat and muscle; therefore, CT is a useful tool for assessing body fat and muscle distributions^9^. This study aimed to evaluate the association between serum testosterone and abdominal body composition based on abdominopelvic CT (APCT) measurements from a large cohort who voluntarily underwent health examinations after adjusting for individual MetS components.

We hypothesized that there are changes in the association between testosterone and abdominal body compositions before and after correction for individual MetS components.

## Materials and methods

### Study design

We used a cross sectional design that is appropriate to confirm the relationship between testosterone and abdominal body composition based on large data from Health Promotion Centre.

### Study participants

**Figure 1:**
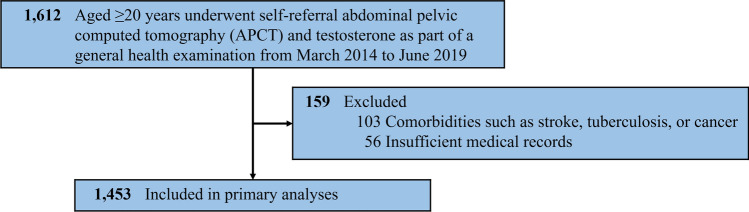
Overview of the study population.

We retrospectively obtained data from 1612 participants aged ≥ 20 years (range: 22–84 years) who underwent self-referral APCT and testosterone level measurement as a part of routine check-ups at the Health Promotion Centre, Ulsan University Hospital, between March 2014 and June 2019. Ulsan is an industrial city in Korea with many large companies, which provide health check-ups every 2 years for their employees. The exclusion criteria were as follows: (1) presence of chronic diseases affecting muscle mass, such as stroke, tuberculosis, chronic kidney disease, chronic liver disease, and cancer, and (2) insufficient medical records. Finally, 1453 subjects were included in the analysis (Fig. 1). Clinical and laboratory variables were collected using the clinical data warehouse platform in conjunction with electronic medical records at the Ulsan University Hospital. This study was approved by Institutional Review Board of Ulsan University Hospital (No. 2021-11-033); it conformed to the principles outlined in the Declaration of Helsinki. The need for informed consent was waived by Institutional Review Board of Ulsan University Hospital owing to the retrospective nature and the anonymization of the data included in the study.

### Clinical and laboratory measurements

Data on clinical factors (e.g., comorbidities, such as hypertension, diabetes, dyslipidemia, and cardiovascular disease) were obtained from systemized self-reported questionnaires issued to the participants prior to their check-up, as described previously^5,6^. Height and weight were collected while the participants wore light clothing without shoes. Body mass index (BMI) was calculated as the weight (kg) divided by the square of the height (m^2^). Waist circumference (cm) was measured midway between the costal margin and the iliac crest at the end of a normal expiration. Blood pressure was checked on the right arm after a 5 or more min rest using an automatic manometer with an appropriate cuff size. After overnight fasting, morning blood samples were collected and were subsequently analyzed at the central laboratory of our hospital. Fasting blood glucose, hemoglobin A1c (HbA1c), albumin, C-reactive protein (CRP), total cholesterol, high-density lipoprotein cholesterol (HDL-C), low-density lipoprotein cholesterol (LDL-C), and triglycerides were measured. CRP was used for analysis as a categorical variable because it was impossible to measure less than 0.042 mg/dL, and the cutoff value was set at 2.0 mg/dL reported in the JUPITER trial^10^. Serum testosterone level was measured by competitive immunoassay using direct chemiluminescent technology on ADVIA Centaur® XP system (Siemens Healthcare Diagnostics, Malvern, PA, USA).

MetS was defined based on the revised National Cholesterol Education Program criteria proposed by the American Heart Association/National Heart, Lung, and Blood Institute^11^. MetS requires the presence of at least three of the following five components: (1) abdominal obesity (waist circumference ≥ 90 cm for Asian men and ≥ 80 cm for Asian women), (2) triglyceride level ≥ 150 mg/dL, (3) HDL cholesterol level < 40 mg/dL for males or < 50 mg/dL for females or those receiving drug treatment, (4) systolic/diastolic blood pressure ≥ 130/85 mmHg or receiving drug treatment, and (5) fasting plasma glucose concentration ≥ 100 mg/dL or receiving drug treatment.

### CT image acquisition and body composition assessment

All CT images were obtained using the SOMATOM Definition Flash system (Siemens Healthcare, Erlangen, Germany), as described previously^5,6^. Enhanced images were obtained after a 80 s delay after contrast injection. The scanning parameters were as follows: beam collimation, 128 × 0.6 mm; beam pitch, 0.6; gantry rotation time, 0.5 s; field of view to fit, 100 kVp. An automatic exposure control system (CARE Dose 4D, Siemens Medical Solutions, Erlangen, Germany) was used.

Body composition was evaluated with APCT using the Asan-J software, which was developed based on ImageJ (NIH, Bethesda, MD, USA), as described previously^5,6,12^. Two consecutive axial CT images at the inferior endplate of the L3 lumbar vertebra were captured for each patient (Appendix S1). Using the Asan-J software, we calculated the total abdominal muscle area (TAMA) (cm^2^), including all muscles in the field (psoas, paraspinal, transversus abdominis, rectus abdominis, quadratus lumborum, and internal/external obliques), with predetermined Hounsfield unit (HU) thresholds on CT. The TAMA was divided into a low-attenuation abdominal muscle area (LAMA) and a normal-attenuation abdominal muscle area (NAMA) based on HUs on CT (TAMA, − 29–150 HU; LAMA, − 29–29 HU; NAMA, 30–150 HU)^13,14^ LAMA implies lipid-rich skeletal muscle, which has more fat elements between and inside the muscle fibers. However, NAMA indicates lipid-poor skeletal muscle, which includes less fat between and inside the muscles^15^. Furthermore, the visceral fat area (VFA) (cm^2^) and the subcutaneous fat area (SFA) (cm^2^) were evaluated using adipose tissue thresholds on CT (− 190 to − 30 HU)^16,17^. We adjusted the cross-sectional areas of the abdominal fat and muscles by BMI based on the Foundation for the National Institutes of Health Sarcopenia Project recommendation^18^; these were named index such as the TAMA index (TAMAi) (TAMAi = TAMA [cm^2^]/BMI [kg/m^2^]), LAMA index (LAMAi) (LAMAi = LAMA [cm^2^]/BMI [kg/m^2^]), NAMA index (NAMAi) (NAMAi = NAMA [cm^2^]/BMI [kg/m^2^]), IMFA index (IMFAi) (IMFAi = IMFAi [cm^2^]/BMI [kg/m^2^]), VFA index (VFAi) (VFAi = VFA [cm^2^]/BMI [kg/m^2^]), and SFA index (SFAi) (SFAi = SFA [cm^2^]/BMI [kg/m^2^]).

### Statistical analyses

Clinical characteristics were summarized as frequency (percentage) for categorical variables and as mean ± standard deviation (SD) for continuous variables. The coefficient of variation was used to show the extent of variability of the continuous variables in this cohort. Before the main analysis, we examined distributions for all continuous variables and found some variables were severely skewed to the right; thus, we conducted log transformation to obtain more stable analysis results (Appendix S2). For example, the testosterone level underwent log_e_-transformation. To determine whether there was an association between testosterone and abdominal body compositions or metabolic factors, we conducted several linear regression model analyses. First, we fitted univariable and multivariable regression models. The effect of abdominal body compositions was then adjusted for important clinical factors such as age, albumin, and five metabolic syndrome components in the multivariable regression analysis. A *p* value less than 0.05 was considered statistically significant (two-tailed). All data analyses were performed using R software version 4.1.2.

## Results

### Participant characteristics and abdominal body composition of study subjects

A total of 1612 subjects were enrolled, of which 159 were excluded because 103 had comorbidities and 56 had insufficient medical records (Fig. 1). The remaining 1453 subjects were included in the primary analysis. The mean age of the study participants was 55.2 ± 8.4 years, and the mean serum testosterone level was 4.36 ± 1.73 ng/ml. Of the 1453 subjects, 450 (31.0%) were diagnosed with MetS. The participant characteristics included abdominal body composition are listed in Table 1.

**Table 1:** Participant characteristics.

Variables	Mean ± SD (n = 1453)	Range
Age, year	55.2 ± 8.4	22–84
BMI, kg/m^2^	24.6 ± 2.9	15.0–47.8
Waist circumference, cm	87.7 ± 7.5	65–150
Systolic blood pressure, mmHg	127 ± 13	90–200
Diastolic blood pressure, mmHg	80 ± 9	50–120
Albumin, g/dL	4.5 ± 0.3	2.7–5.4
Aspartate transaminase, IU/L	27.3 ± 14.9	11–228
Alanine aminotransferase, IU/L	32.2 ± 20.2	7–270
Blood urea nitrogen, mg/dL	13.9 ± 3.5	5.3–34.7
Creatinine, mg/dL	0.9 ± 0.2	0.5–2.1
Hemoglobin A1c, %	5.8 ± 0.9	4.3–12.3
Triglyceride, mg/dL	130 ± 90	22–822
Total cholesterol, mg/dL	185 ± 38	63–346
High-density lipoprotein cholesterol, mg/dL	48.5 ± 13.6	18–130
Low-density lipoprotein cholesterol, mg/dL	127.2 ± 35.6	27–291
Fasting blood glucose, mg/dL	100 ± 24	56–255
**CRP, mg/dL, n (%)**		
≥ 2.0	23 (1.6)	
< 2.0	1196 (82.4)	
Missing data	218 (15.0)	
Metabolic syndrome, n (%)	450 (31.0)	
Waist circumference ≥ 90 cm, n (%)	490 (33.7)	
Triglyceride ≥ 150 mg/dL, n (%)	413 (28.4)	
High-density lipoprotein cholesterol < 40 mg/dL or receiving drug treatment, n (%)	433 (29.8)	
Systolic blood pressure ≥ 130/85 mmHg or receiving drug treatment, n (%)	910 (62.6)	
Fasting blood glucose ≥ 100 mg/dL or receiving drug treatment, n (%)	498 (34.3)	
**Abdominal body composition derived from computed tomography**		
Subcutaneous fat area, cm^2^	131 ± 54	1–859
Subcutaneous fat index, cm^2^/(kg/m^2^)	5.20 ± 1.67	0.06–17.97
Visceral fat area, cm^2^	139 ± 63	2–422
Visceral fat index, cm^2^/(kg/m^2^)	5.53 ± 2.17	0.12–14.03
Intermuscular fat area, cm^2^	5.8 ± 3.8	0–33
Intermuscular fat index, cm^2^/(kg/m^2^)	0.23 ± 0.14	0.00–1.23
Total abdominal muscle area, cm^2^	157 ± 23	93–229
Total abdominal muscle index, cm^2^/(kg/m^2^)	6.41 ± 0.70	3.28–8.96
Normal-attenuation muscle area, cm^2^	128 ± 22	58–203
Normal-attenuation muscle index, cm^2^/(kg/m^2^)	5.21 ± 0.83	2.11–8.29
Low-attenuation muscle area, cm^2^	30 ± 11	7–92
Low-attenuation muscle index, cm^2^/(kg/m^2^)	1.19 ± 0.38	0.35–3.09

*SD* Standard deviation.

### Association between testosterone and abdominal body composition

**Figure 2:**
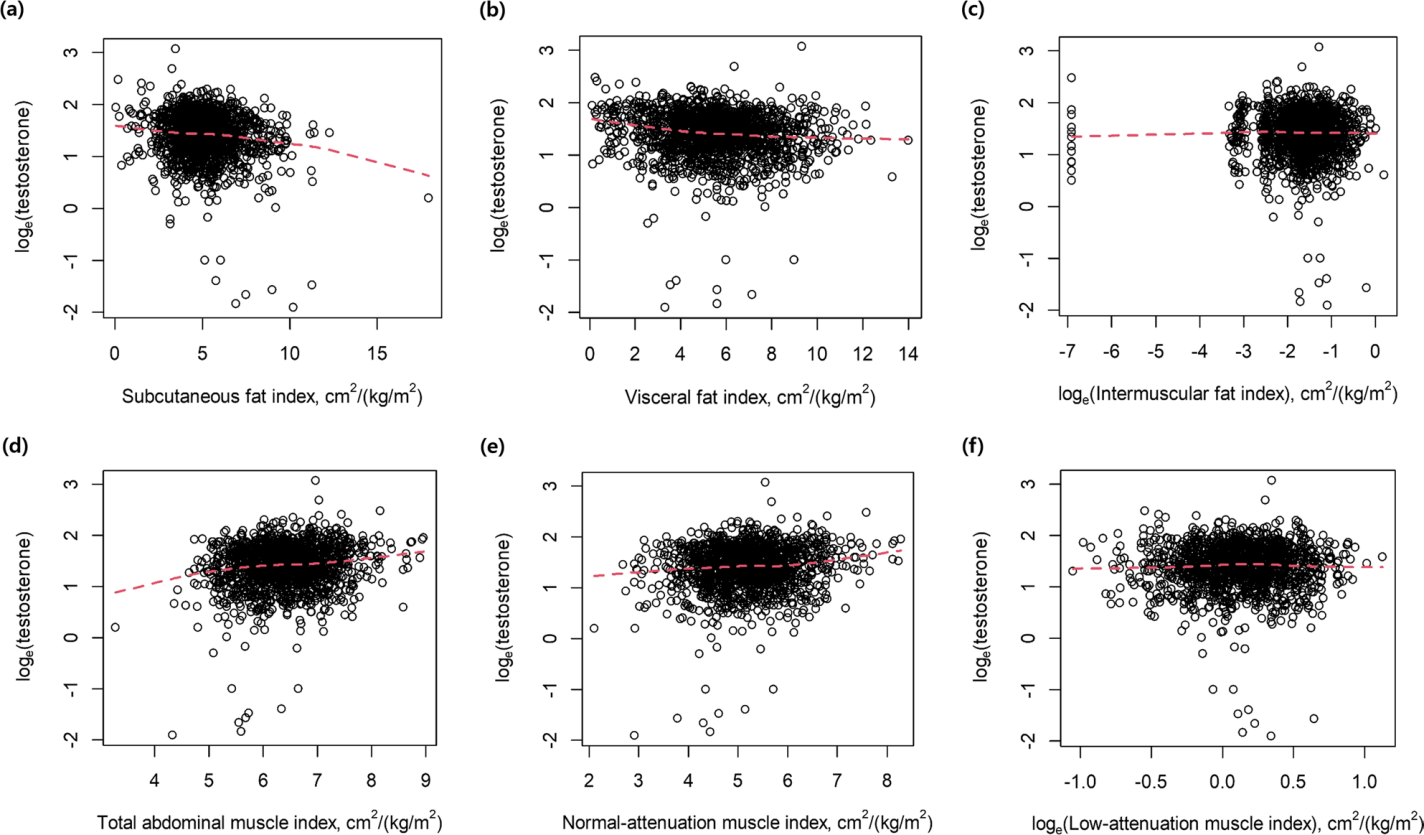
Scatter plots to show the association between log_e_-transformed testosterone and individual body composition parameters. (**a**) subcutaneous fat index, (**b**) visceral fat index, (**c**) intermuscular fat index, (**d**) total abdominal muscle index, (**e**) normal-attenuation muscle index, and (**f**) log_e_ (low-attenuation muscle index).

In the univariable linear regression analyses, the log_e_-transformed testosterone had a negative correlation with HbA1c (unstandardized β (95% confidence interval [CI]) = − 0.060 (− 0.086 to − 0.034), *p* < 0.001) and CRP ≥ 2.0 mg/dL (unstandardized β (95% CI) = − 0.381 (− 0.572 to − 0.191), *p* < 0.001) (Table 2 and Fig. 2). All metabolic components had a negative correlation with the log_e_-transformed testosterone. Regarding abdominal body composition, SFAi (unstandardized β (95% CI) = − 0.048 (− 0.059 to − 0.031), *p* < 0.001), VFAi (unstandardized β (95% CI) = − 0.026 (− 0.037 to − 0.016), *p* < 0.001), TAMAi (unstandardized β (95% CI) = 0.110 (0.077–0.142), *p* < 0.001), and NAMAi (unstandardized β (95% CI) = 0.076 (0.049–0.104), *p* < 0.001) had an association with the log_e_-transformed testosterone.

**Table 2:** Univariable linear regression analysis to determine the relationship with log_e_-transformed testosterone.

Variables	Coefficient (β)	95% CI		*P* value
		LB	UB	
Age, year	0.001	− 0.001	0.004	0.310
Albumin, g/dL	− 0.046	− 0.115	0.023	0.193
Hemoglobin A1c, %	− 0.060	− 0.086	− 0.034	< 0.001
CRP (≥ 2.0 mg/dL vs. < 2.0 mg/dL)	− 0.381	− 0.572	− 0.191	< 0.001
**Metabolic components**				
Waist circumference ≥ 90 cm (yes vs. no)	− 0.117	− 0.166	− 0.068	< 0.001
Triglyceride ≥ 150 mg/dL (yes vs. no)	− 0.070	− 0.122	− 0.019	0.008
High-density lipoprotein cholesterol < 40 mg/dL or receiving drug treatment (yes vs. no)	− 0.117	− 0.168	− 0.066	< 0.001
Systolic blood pressure ≥ 130/85 mmHg or receiving drug treatment (yes vs. no)	− 0.078	− 0.126	− 0.030	0.002
Fasting blood glucose ≥ 100 mg/dL or receiving drug treatment (yes vs. no)	− 0.110	− 0.159	− 0.061	< 0.001
**Abdominal body composition from CT scan**				
Subcutaneous fat index, cm^2^/(kg/m^2^)	− 0.048	− 0.059	− 0.031	< 0.001
Visceral fat index, cm^2^/(kg/m^2^)	− 0.026	− 0.037	− 0.016	< 0.001
Log_e_ (Intermuscular fat index), cm^2^/(kg/m^2^)	− 0.011	− 0.040	0.018	0.464
Total abdominal muscle index, cm^2^/(kg/m^2^)	0.110	0.077	0.142	< 0.001
Normal-attenuation muscle index, cm^2^/(kg/m^2^)	0.076	0.049	0.104	< 0.001
Log_e_ (Low-attenuation muscle index), cm^2^/(kg/m^2^)	0.013	− 0.061	0.086	0.737

*CI* Confidence interval, *LB* Lower bound, *UB* Upper bound, *CT* Computed tomography.

After adjustment for age, individual metabolic components, albumin, hemoglobin A1c, and CRP ≥ 2 mg/dL, SFAi (unstandardized β (95% CI) = − 0.042 (− 0.059 to − 0.024), *p* < 0.001), TAMAi (unstandardized β (95% CI) = 0.115 (0.076–0.153), *p* < 0.001), NAMAi (unstandardized β (95% CI) = 0.070 (0.035–0.104), *p* < 0.001), and log_e_-transformed LAMAi (unstandardized β (95% CI) = 0.140 (0.050–0.230), *p* = 0.002) had an association with the log_e_-transformed testosterone (Table 3 and appendix S3).

**Table 3:** Multivariable linear regression analysis to determine the relationship with log_e_-transformed testosterone (abdominal body composition).

Variables	Coefficient (β)	95% CI		*P* value
		LB	UB	
Subcutaneous fat index, cm^2^/(kg/m^2^)	− 0.042	− 0.059	− 0.024	< 0.001
Visceral fat index, cm^2^/(kg/m^2^)	− 0.005	− 0.020	0.009	0.483
Log_e_ (Intermuscular fat index), cm^2^/(kg/m^2^)	0.019	− 0.020	0.058	0.338
Total abdominal muscle index, cm^2^/(kg/m^2^)	0.115	0.076	0.153	< 0.001
Normal-attenuation muscle index, cm^2^/(kg/m^2^)	0.070	0.035	0.104	< 0.001
Log_e_ (Low-attenuation muscle index), cm^2^/(kg/m^2^)	0.140	0.050	0.230	0.002

*CI* Confidence interval, *LB* Lower bound, *UB* Upper bound.

Covariates in the multivariate model include age, individual metabolic factors, albumin, hemoglobin A1c, and C-reactive protein ≥ 2 mg/dL.

## Discussion

This study provided further evidence of the very close relationship between testosterone and abdominal body composition, including abdominal fat and muscle. However, as shown in previous studies and our study, testosterone is also closely related to MetS^7,19^. In other words, low testosterone was highly correlated with the prevalence of MetS. In addition, men with decreased testosterone were closely related to increased insulin resistance and type 2 diabetes prevalence^20^. This was also reported in the Men Androgen Inflammation Lifestyle Environment and Stress study^21^. This is problematic because, as previous studies have shown, MetS and glycemic status are highly correlated with abdominal body composition^5,12^. The effect of testosterone on abdominal body composition could be secondary effects of MetS because of the relationship between testosterone and MetS. A previous study demonstrated this relationship between MetS and low testosterone in men, especially when visceral adiposity is high^19^. To date, there has been no study on the relationship between testosterone and abdominal body composition after adjusting for MetS as a confounding factor. Allan et al.^4^ reported the results of a randomized controlled trial (RCT) that confirmed a change in abdominal body composition after the administration of testosterone replacement therapy in a group matched with MetS. However, since this study did not evaluate the improvement in MetS due to testosterone replacement for 52 weeks, it was challenging to say that the analysis was done after adjusting MetS accurately.

In this study, after adjusting for MetS, testosterone only correlated with subcutaneous fat but not with visceral fat. The result that testosterone was related to subcutaneous fat but not to visceral fat was similarly reported in two RCTs^22,23^. These results had also been proven in animal experiments^24^. However, most previous studies performed the analysis without adjusting for MetS, showing a close relationship between testosterone and visceral fat, unlike the results in the present study^4,25,26^. MetS itself is not related to subcutaneous fat and is closely associated with visceral fat^27–29^. Therefore, the result could be different if the direct relationship between testosterone and abdominal body composition is analyzed after adjusting for MetS. Even in this study, before adjusting for MetS, testosterone was found to be closely related to both subcutaneous and visceral fat. These results can be explained using testosterone metabolites^30^. Testosterone is converted to estradiol by aromatase and dihydrotestosterone (DHT) by 5α-reductase. DHT specifically impedes subcutaneous fat growth, whereas estradiol specifically prevents the expansion of visceral fat. Therefore, the inhibition of estradiol was unable to prevent visceral fat growth^31^. Most circulating estradiol is aromatized from testosterone predominantly in adipose tissue. Therefore, if individual MetS components including obesity are adjusted, estradiol could also be adjusted, so that only the effect of DHT between testosterone metabolites would remain. Hence, it is considered that testosterone was related only to subcutaneous fat when individual MetS components were adjusted.

Testosterone is closely related to abdominal muscle mass. Our study also showed a positive correlation between testosterone and abdominal muscle mass, which was also observed in studies reporting changes in lean body mass or muscle size after testosterone replacement therapy^32–34^. Many studies have reported the mechanisms of the effect of testosterone on muscle; testosterone enhances an increase in muscle fiber size by increased protein synthesis, stimulates the mitotic activity of satellite cell in myoblast culture systems, and increases IGF-1 expression^35^. Most previous studies reported that only fat-free mass was associated with testosterone^33,35^. Similar to other studies, fat-rich muscle was not related to testosterone in the univariate analysis in this study. A previous our study showed that MetS was significantly associated with fat-rich muscle in males (OR 1.771, *p* < 0.001)^12^. Therefore, in order to analyze the relationship between testosterone and abdominal muscle mass, it was necessary to adjust for MetS. Interestingly, testosterone had a positive correlation with muscle regardless of qualitative features such as fat-rich and fat–free. This result could be because testosterone correlated positively with the fat-rich muscle, whereas MetS correlated negatively.

Our study had several limitations. First, since this study was designed as a cross-sectional study, we could not clarify the causal relationship between testosterone and abdominal body composition. Therefore, prospective randomized controlled trials are required to confirm the exact causal relationship. Second, the study was performed in a single health promotion center and most of the subjects were relatively healthy. Thus, there is a possibility of selection bias. To minimize the bias, we tried to include a relatively large sample size and to perform a multivariable linear regression analysis. Third, Ideally, sex hormone binding globulin should have been assessed and calculated free and bioavailable testosterone derived as two measurements reflect biologically active testosterone values as SHBG bound testosterone is considered inactive. However, Many subjects did not check SHBG and free testosterone because all subjects were tested for the health check.

## Conclusions

Our study identified the necessity of adjusting for MetS to confirm the association between testosterone level and abdominal body composition. After adjusting for individual MetS components, testosterone was significantly associated with subcutaneous fat, but not visceral fat. In addition, testosterone had a positive correlation with abdominal muscle regardless of qualitative features such as fat-rich and fat-free. These results suggest that testosterone has a close relationship with subcutaneous fat and abdominal muscle, regardless of MetS.

## Supplementary Information

Supplementary Information.

## Data Availability

Seong Cheol Kim had full access to all the data in the study and takes responsibility for the integrity of the data and the accuracy of the data analysis.

## Abbreviations

MetS: Metabolic syndrome
CT: Computed tomography
APCT: Abdominopelvic computed tomography
BMI: Body mass index
HbA1c: Hemoglobin A1c
CRP: C-reactive protein
HDL-C: High-density lipoprotein cholesterol
LDL-C: Low-density lipoprotein cholesterol
HU: Hounsfield unit
TAMA: Total abdominal muscle area
TAMAi: Total abdominal muscle area index
LAMA: Low-attenuation abdominal muscle area
LAMAi: Low-attenuation abdominal muscle area index
NAMA: Normal-attenuation abdominal muscle area
NAMAi: Low-attenuation abdominal muscle area index
VFA: Visceral fat area
VFAi: Visceral fat area index
SFA: Subcutaneous fat area
SFAi: Subcutaneous fat area index
SD: Standard deviation
CV: Coefficient of variation
CI: Confidence interval
DHT: Dihydrotestosterone
