# Supplementary material for: Testosterone is associated with abdominal body composition derived from computed tomography: a large cross sectional study
Source: Sci Rep. 2022 Dec 29;12:22528. doi: 10.1038/s41598-022-27182-y (PMC9800400; doi:10.1038/s41598-022-27182-y)
Supplement: Supplementary file 1 — Supplementary Information. [file 41598_2022_27182_MOESM1_ESM.docx]

**Appendix S1**

Figure. Segmental abdominal body fat and muscle analysis at the L3 vertebra on abdominopelvic computed tomography. HU: Hounsfield units; SFA: subcutaneous fat area; VFA: visceral fat area; IMFA: intermuscular fat area; LAMA: low-attenuation abdominal muscle area; NAMA: normal-attenuation muscle area; TAMA: total abdominal muscle area

**
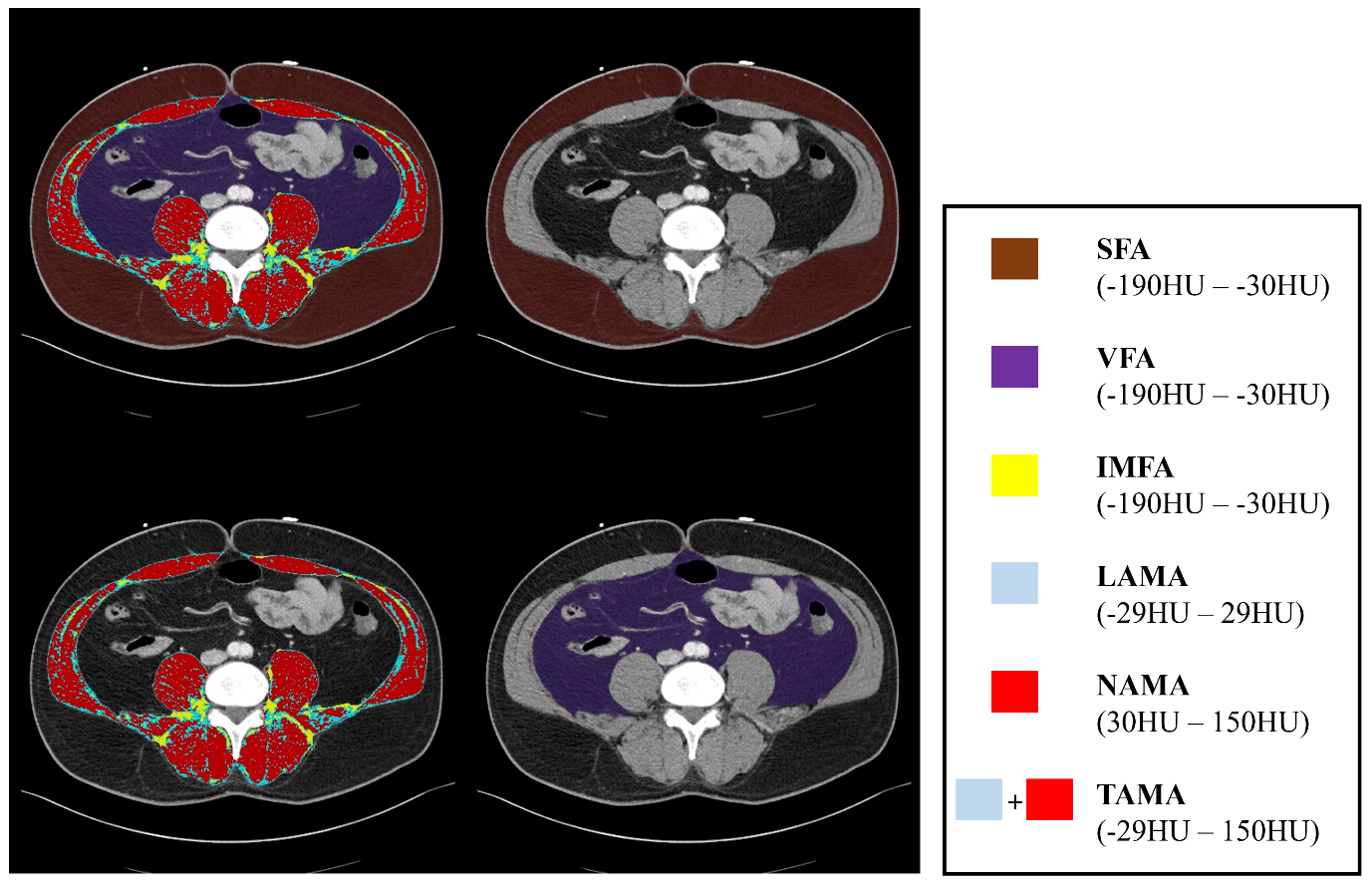
**

**Appendix S2**

Figure. Distribution of (a) testosterone and (b) loge-transformed testosterone in the cohort.


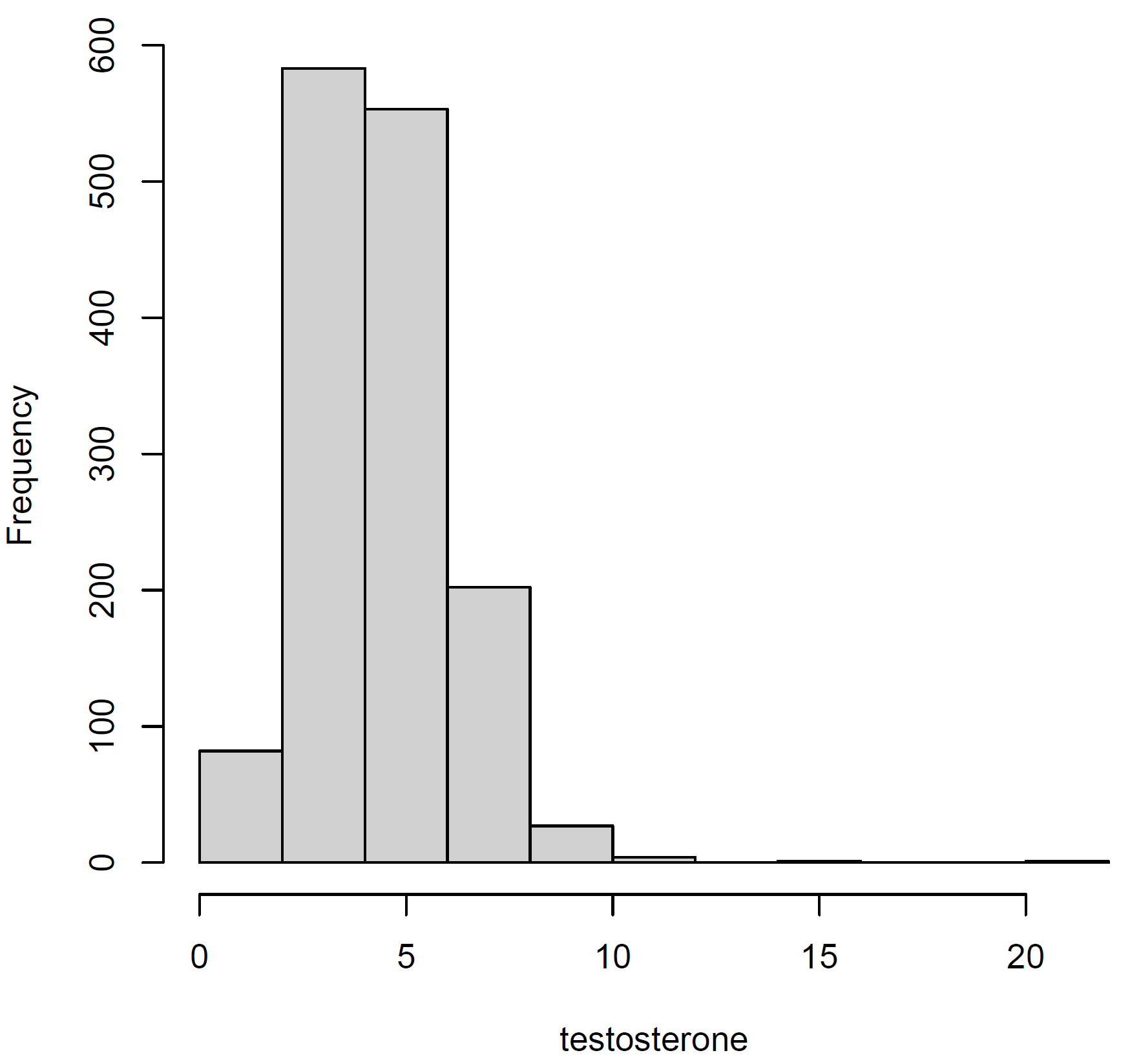


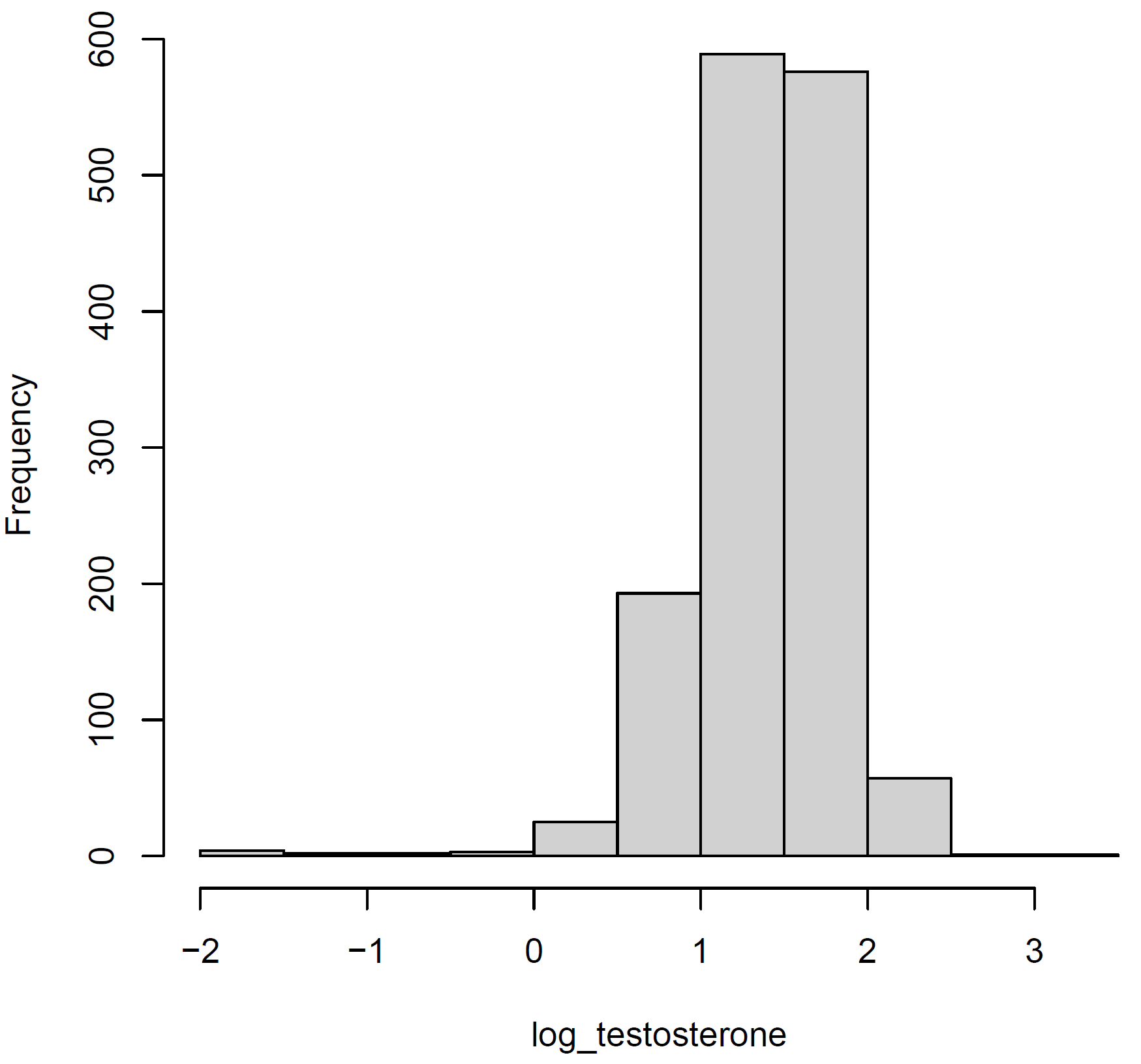


**Appendix S3**

Multivariable analysis (each factors)

1. Subcutaneous fat area index

| Variables | Coefficient  (β) | 95% CI | | P-value |
| --- | --- | --- | --- | --- |
|  |  | LB | UB |  |
| Age, year | -0.001 | -0.004 | 0.003 | 0.727 |
| CRP (≥2.0 vs. <2.0) | -0.417 | -0.605 | -0.229 | <0.001 |
| Albumin, g/dL | -0.114 | -0.193 | -0.035 | 0.005 |
| Hemoglobin A1c, % | -0.049 | -0.082 | -0.016 | 0.004 |
| Waist circumference ≥90 cm (yes vs. no) | -0.029 | -0.087 | 0.029 | 0.328 |
| Triglyceride ≥150 mg/dL (yes vs. no) | 0.017 | -0.042 | 0.076 | 0.570 |
| High-density lipoprotein <40 mg/dL or receiving drug treatment (yes vs. no) | -0.092 | -0.148 | -0.036 | 0.001 |
| Systolic blood pressure ≥130/85 mmHg or receiving drug treatment (yes vs. no) | -0.058 | -0.111 | -0.005 | 0.032 |
| Fasting blood glucose ≥100 mg/dL or receiving drug treatment (yes vs. no) | -0.033 | -0.096 | 0.031 | 0.310 |
| **Subcutaneous fat index, cm2/kg/m2** | **-0.042** | **-0.059** | **-0.024** | **<0.001** |

CI = confidence interval

LB = lower bound

UB = upper bound

1. Visceral fat area index

| Variables | Coefficient  (β) | 95% CI | | P-value |
| --- | --- | --- | --- | --- |
|  |  | LB | UB |  |
| Age, year | 0.001 | -0.002 | 0.005 | 0.374 |
| CRP (≥2.0 vs. <2.0) | -0.415 | -0.604 | -0.226 | <0.001 |
| Albumin, g/dL | -0.121 | -0.201 | -0.041 | 0.003 |
| Hemoglobin A1c, % | -0.041 | -0.074 | -0.008 | 0.015 |
| Waist circumference ≥90 cm (yes vs. no) | -0.073 | -0.133 | -0.013 | 0.017 |
| Triglyceride ≥150 mg/dL (yes vs. no) | 0.025 | -0.035 | 0.085 | 0.416 |
| High-density lipoprotein <40 mg/dL or receiving drug treatment (yes vs. no) | -0.093 | -0.150 | -0.036 | 0.001 |
| Systolic blood pressure ≥130/85 mmHg or receiving drug treatment (yes vs. no) | -0.061 | -0.115 | -0.007 | 0.026 |
| Fasting blood glucose ≥100 mg/dL or receiving drug treatment (yes vs. no) | -0.042 | -0.106 | 0.022 | 0.200 |
| **Visceral fat index, cm2/kg/m2** | -0.005 | -0.020 | 0.009 | 0.483 |

CI = confidence interval

LB = lower bound

UB = upper bound

1. Log_e_ (Intramuscular fat index)

| Variables | Coefficient  (β) | 95% CI | | P-value |
| --- | --- | --- | --- | --- |
|  |  | LB | UB |  |
| Age, year | 0.001 | -0.002 | 0.004 | 0.526 |
| CRP (≥2.0 vs. <2.0) | -0.410 | -0.600 | -0.221 | <0.001 |
| Albumin, g/dL | -0.124 | -0.203 | -0.044 | 0.002 |
| Hemoglobin A1c, % | -0.043 | -0.073 | -0.010 | 0.012 |
| Waist circumference ≥90 cm (yes vs. no) | -0.088 | -0.143 | -0.033 | 0.002 |
| Triglyceride ≥150 mg/dL (yes vs. no) | 0.020 | -0.039 | 0.079 | 0.515 |
| High-density lipoprotein <40 mg/dL or receiving drug treatment (yes vs. no) | -0.093 | -0.150 | -0.036 | 0.001 |
| Systolic blood pressure ≥130/85 mmHg or receiving drug treatment (yes vs. no) | -0.064 | -0.118 | -0.011 | 0.019 |
| Fasting blood glucose ≥100 mg/dL or receiving drug treatment (yes vs. no) | -0.042 | -0.106 | 0.022 | 0.200 |
| **Log_e_ (Intramuscular fat index), cm2/kg/m2** | 0.019 | -0.020 | 0.058 | 0.338 |

CI = confidence interval

LB = lower bound

UB = upper bound

1. Total abdominal muscle index

| Variables | Coefficient  (β) | 95% CI | | P-value |
| --- | --- | --- | --- | --- |
|  |  | LB | UB |  |
| Age, year | 0.004 | 0.001 | 0.007 | 0.014 |
| CRP (≥2.0 vs. <2.0) | -0.392 | -0.579 | -0.205 | <0.001 |
| Albumin, g/dL | -0.108 | -0.186 | -0.029 | 0.007 |
| Hemoglobin A1c, % | -0.035 | -0.068 | -0.003 | 0.034 |
| Waist circumference ≥90 cm (yes vs. no) | -0.061 | -0.114 | -0.007 | 0.028 |
| Triglyceride ≥150 mg/dL (yes vs. no) | 0.021 | -0.038 | 0.079 | 0.488 |
| High-density lipoprotein <40 mg/dL or receiving drug treatment (yes vs. no) | -0.087 | -0.144 | -0.031 | 0.002 |
| Systolic blood pressure ≥130/85 mmHg or receiving drug treatment (yes vs. no) | -0.051 | -0.104 | 0.001 | 0.057 |
| Fasting blood glucose ≥100 mg/dL or receiving drug treatment (yes vs. no) | -0.048 | -0.111 | 0.015 | 0.135 |
| **Total abdominal muscle index, cm2/kg/m2** | 0.115 | 0.076 | 0.153 | <0.001 |

CI = confidence interval

LB = lower bound

UB = upper bound

1. Normal-attenuation muscle index

| Variables | Coefficient  (β) | 95% CI | | P-value |
| --- | --- | --- | --- | --- |
|  |  | LB | UB |  |
| Age, year | 0.003 | 0.000 | 0.007 | 0.040 |
| CRP (≥2.0 vs. <2.0) | -0.397 | -0.586 | -0.209 | <0.001 |
| Albumin, g/dL | -0.127 | -0.206 | -0.047 | 0.002 |
| Hemoglobin A1c, % | -0.037 | -0.070 | -0.004 | 0.030 |
| Waist circumference ≥90 cm (yes vs. no) | -0.053 | -0.109 | 0.003 | 0.062 |
| Triglyceride ≥150 mg/dL (yes vs. no) | 0.024 | -0.034 | 0.083 | 0.417 |
| High-density lipoprotein <40 mg/dL or receiving drug treatment (yes vs. no) | -0.092 | -0.149 | -0.036 | 0.001 |
| Systolic blood pressure ≥130/85 mmHg or receiving drug treatment (yes vs. no) | -0.053 | -0.106 | 0.000 | 0.052 |
| Fasting blood glucose ≥100 mg/dL or receiving drug treatment (yes vs. no) | -0.047 | -0.110 | 0.017 | 0.150 |
| **Normal-attenuation muscle index, cm2/kg/m2** | 0.070 | 0.035 | 0.104 | <0.001 |

CI = confidence interval

LB = lower bound

UB = upper bound

1. Log_e_ (Low-attenuation muscle index)

| Variables | Coefficient  (β) | 95% CI | | P-value |
| --- | --- | --- | --- | --- |
|  |  | LB | UB |  |
| Age, year | 0.000 | -0.003 | 0.003 | 0.872 |
| CRP (≥2.0 vs. <2.0) | -0.415 | -0.603 | -0.226 | <0.001 |
| Albumin, g/dL | -0.104 | -0.185 | -0.024 | 0.011 |
| Hemoglobin A1c, % | -0.045 | -0.078 | -0.012 | 0.007 |
| Waist circumference ≥90 cm (yes vs. no) | -0.109 | -0.165 | -0.052 | <0.001 |
| Triglyceride ≥150 mg/dL (yes vs. no) | 0.014 | -0.044 | 0.073 | 0.631 |
| High-density lipoprotein <40 mg/dL or receiving drug treatment (yes vs. no) | -0.090 | -0.147 | -0.033 | 0.002 |
| Systolic blood pressure ≥130/85 mmHg or receiving drug treatment (yes vs. no) | -0.068 | -0.121 | -0.014 | 0.013 |
| Fasting blood glucose ≥100 mg/dL or receiving drug treatment (yes vs. no) | -0.041 | -0.105 | 0.022 | 0.204 |
| **Log_e_ (Low-attenuation muscle index), cm2/kg/m2** | 0.140 | 0.050 | 0.230 | 0.002 |

CI = confidence interval

LB = lower bound

UB = upper bound
